# Live attenuated coronavirus vaccines deficient in N7-Methyltransferase activity induce both humoral and cellular immune responses in mice

**DOI:** 10.1080/22221751.2021.1964385

**Published:** 2021-08-18

**Authors:** Zhen Zhang, Qianyun Liu, Ying Sun, Jiali Li, Jiejie Liu, Ruangang Pan, Liu Cao, Xianying Chen, Yingjian Li, Yuzhen Zhang, Ke Xu, Deyin Guo, Li Zhou, Ke Lan, Yu Chen

**Affiliations:** a State Key Laboratory of Virology, Modern Virology Research Center, Institute for Vaccine Research, RNA Institute, College of Life Sciences, Wuhan University, Wuhan, People’s Republic of China; b School of Chinese Medicine (Zhongjing School), Henan Univesity of Chinese Medicne, Zhengzhou, People’s Republic of China; c Center for Infection & Immunity Study, School of Medicine, Sun Yat-sen University, Guangzhou, People’s Republic of China; d Animal Bio-Safety Level III Laboratory at Center for Animal Experiments, Wuhan University School of Medicine, Wuhan, People’s Republic of China

**Keywords:** Coronavirus, vaccine, N7-Methyltransferase, nsp14, cap structure

## Abstract

Coronaviruses (CoVs) can infect a variety of hosts, including humans, livestock and companion animals, and pose a serious threat to human health and the economy. The current COVID-19 pandemic, which is caused by severe acute respiratory syndrome coronavirus 2 (SARS-CoV-2), has killed millions of people. Unfortunately, effective treatments for CoVs infection are still lacking, suggesting the importance of coronavirus vaccines. Our previous work showed that CoV nonstuctural protein 14 (nsp14) functions as (guanine-N7)-methyltransferase (N7-MTase), which is involved in RNA cap formation. Moreover, we found that N7-MTase is well conserved among different CoVs and is a universal target for developing antivirals against CoVs. Here, we show that N7-MTase of CoVs can be an ideal target for designing live attenuated vaccines. Using murine hepatitis virus strain A59 (MHV-A59), a representative and well-studied model of coronaviruses, we constructed N7-MTase-deficient recombinant MHV D330A and Y414A. These two mutants are highly attenuated in mice and exhibit similar replication efficiency to the wild-type (WT) virus in the cell culture. Furthermore, a single dose immunization of D330A or Y414A can induce long-term humoral immune responses and robust CD4^+^ and CD8^+^ T cell responses, which can provide full protection against the challenge of a lethal-dose of MHV-A59. Collectively, this study provides an ideal strategy to design live attenuated vaccines for coronavirus by abolishing viral RNA N7-MTase activity. This approach may apply to other RNA viruses that encode their own conservative viral N7-methyltransferase.

## Introduction

Coronaviruses are enveloped positive-sense single-stranded RNA viruses. They can infect different hosts, including humans, livestock, mice, bats and other wild animals, and pose a serious threat to human health, public safety and the economy [1]. According to the International Committee on Taxonomy of Viruses (ICTV) classification, CoVs belong to subfamily Coronavirinae in the family Coronaviridae of the order Nidovirales [2]. Coronavirinae is further subdivided into four genera: *Alphacoronavirus*, *Betacoronavirus*, *Gammacoronavirus* and *Deltacoronavirus*. The former two infect only mammals, while the latter two primarily infect birds [3]. Since the early 1930s, a variety of domestic diseases have been found related to CoV infections. For instance, infectious bronchitis virus (IBV) usually causes avian infectious bronchitis accompanied by upper respiratory symptoms and urinary and reproductive system failures, resulting in low production rates, egg drop, and death in chickens [4]. Several porcine CoVs, such as transmissible gastroenteritis virus (TGEV), porcine respiratory coronavirus (PRCV), porcine epidemic diarrhoea virus (PEDV), porcine deltacoronavirus (PDCoV), and emerging swine acute diarrhoea syndrome-coronavirus (SADS-CoV), have widely circulated in pigs worldwide and caused severe gastrointestinal diseases in suckling piglets with high mortality, culminating in major losses to the pork industry [5,6]. Simultaneously, MHV, a betacoronavirus that infects mice and induces disease in several organ systems, was widely studied to understand the pathogenic biology of coronavirus [7]. In humans, some low pathogenic CoVs, such as HCoV-229E and HCoV-OC43, are widely spread among people but primarily cause self-limiting disease with mild symptoms [8]. Remarkably, three highly transmissible and pathogenic zoonotic betacoronaviruses, namely, severe acute respiratory syndrome coronavirus (SARS-CoV), Middle East respiratory syndrome coronavirus (MERS-CoV), and SARS-CoV-2 have emerged in humans over the last two decades [9–13]. Infection with these viruses can cause fatal respiratory illness, making emerging coronaviruses a new public health concern [14]. SARS-CoV and MERS-CoV caused local outbreaks with high morbidity and mortality in 2003 and 2012, respectively, whereas SARS-CoV-2 has resulted in the COVID-19 pandemic [14,15]. As of June 2021, there have been 170 million confirmed cases of COVID-19, including at least 3.69 million deaths according to the latest report from the WHO (https://covid19.who.int/).

Since the outbreak of the emerging coronavirus, much progress has been made on coronavirus biology. The coronavirus virion consists of four structural proteins – spike (S), envelope (E), membrane (M), nucleocapsid (N) – and a positive-sense single-stranded RNA (+ssRNA) (≈30 kb) genome with a 5′-cap structure and 3′-poly-A tail encapsulated in the virus particle. Two-thirds of the 5′-genome contains open reading frame 1a (ORF1a) and ORF1b, and can be directly translated into two large polyproteins, 1a/1ab (pp1a/pp1ab), which are cleaved into 15 or 16 non-structural proteins (nsp) to form the replication-transcription complex (RTC) in double-membrane vesicles (DMVs) [1,16–18]. The remaining third of the 3′-genome encodes structural and virus-specific accessory proteins, which are expressed from subgenomic RNAs (sgRNAs) synthesized through the discontinuous viral transcription process [3,19,20]. During the viral life cycle, CoVs primarily enter cells through the interaction between S protein and specific receptors. They subsequently utilize cellular machinery to translate their nsps, which conduct replication of genomic RNA and transcription of sgRNAs to produce full-length copies and viral proteins [3]. It is noteworthy that the mature 5′-cap structure, which harbours methylation at the N7 position of the capping guanylate and 2′-O position of the first and second nucleotides, is important for RNA stability and efficient translation during the replication of CoVs [21]. Besides, coronavirus ribose 2′-O-methylation allows the viral RNA to evade the recognition of host RNA sensors, such as Mda5, thus perturbing the host’s innate immune responses [22]. For CoVs, cap formation requires the following enzymes: RNA triphosphatase (TPase), RNA guanylyltransferase (GTase), N7-MTase, and 2′-O-MTase. We previously showed that CoV nsp14 functions as N7-MTase to produce a cap-0 structure, which is the basis of the cap-1 structure that mediates immune escape [22,23]. Moreover, we showed that the N7-MTase is well conserved among different CoVs, and is a universal target for developing antivirals against CoVs [24,25]. Meanwhile, the critical amino acid residues essential for N7-MTase activity were also identified. However, the details of different residues on viral replication and pathogenicity are still not well understood.

Here, we introduced single amino acid substitution at conserved position D330 or Y414 of MHV nsp14 and generated two MHV N7-MTase mutant virus D330A and Y414A by a reverse genetic system. We found that the two mutants were genetically stable and slightly attenuated in the cell culture, but were extremely attenuated in virulence and pathogenicity in mice. Furthermore, we showed that a single dose of D330A or Y414A immunization could elicit long-term humoral immune responses that last for at least 48 weeks. More importantly, the immunization could also induce robust CD4^+^ and CD8^+^ T cell responses and provide full protection against lethal challenge with WT MHV-A59 strains in mice. This study demonstrates the feasibility of using N7-MTase as a target to design live attenuated vaccines. Considering the conservation of N7-MTase in different CoVs, this strategy is promising for applications to develop live attenuated vaccines against new emerging CoVs, especially CoVs circulating in livestock.

## Materials and methods

### Cells and animals

CV-1, BHK-21-MHV-N, HeLa-D980R, 17CL-1, L2 and Neuro 2a cells were cultured at 37°C with 5% CO_2_ in Dulbecco modified Eagle’s medium (DMEM, Gibco) supplemented with 10% foetal bovine serum (FBS, Gbico) and 1% penicillin/streptomycin (Gbico). Four-week-old SPF C57BL/6 mice were purchased from Beijing Vital River Laboratory Animal Technology Co. Ltd. (Beijing, China). All mice were maintained in individually ventilated cages (IVC) in an animal biosafety level 2 (ABSL-2) facility and received care in compliance with international legal requirements throughout the experiments.

### Protein expression and purification

The recombinant plasmids encoding MHV nsp14, MHV nsp14 mutants or SARS-CoV-2 nsp14 were constructed as previously described [24]. They were then transformed into *Escherichia coli* BL21. Cells were cultivated at 37°C in a shaker incubator and induced with 0.4 mM isopropyl β-D-thiogalactoside (IPTG) when OD_600_ nm reached 0.8. Cells were harvested after induction at 16°C for 16 h, and lysed by enzymolysis in 50 mM NaH_2_PO_4_ (pH 8.0), 300 mM NaCl, 10 mM imidazole, 5% (vol/vol) glycerol, 0.4 mg/mL lysosome, 1 mM PMSF, and 0.02% (v/v) NP40, and DNAs and RNAs in the lysate were digested by 10 mM MgCl_2_, 5 μg/mL DNase I and 10 μg/mL RNase A. After centrifugation, recombinant protein was purified using Ni-affinity resin (Novagen) with different concentrations of imidazole for washing and elution, which was followed by chromatography using a Superdex 200 column of AKTA pure GE. All nsp14 mutants were expressed and purified as described above.

### N7-MTase activity assay

RNA (5′-GATTTAAGTGAATAGCTTGG-3′) was synthesized in vitro (RiboMAX™ Large Scale RNA Production System-T7, Promega) and then capped by vaccine capping system (NEB). Purified proteins and 1 μM 5′ capping RNA substrate were added to a reaction system which contained 1 mM ^3^H-SAM, 40 mM Tris (pH 8.0), 5 mM DTT, and 1U RNase inhibitor. After being incubated at 37°C for 1 h, the RNA was purified by DEAE Sephadex A-25 (GE) and added to a liquid scintillation cocktail to test its radioactivity by Liquid Scintillation Analyzer (PerkinElmer).

### Construction of MHV-A59 N7-MTase mutants

Recombinant vaccinia virus inf-1 (vMHV-inf-1) containing a cloned, full-length MHV-A59 cDNA and recombinant vaccinia virus vMHV-nsp14-gpt-in, in which the MHV-A59 nsp14 gene was replaced by the *E. coli* guanine-phosphoribosyl-transferase (GPT) gene were kindly provided by Prof. Chang Guohui. The MHV-A59 nsp14 gene (nucleotides 17,634–17,660) was cloned from vMHV-inf-1 DNA by standard PCR, and nsp14 mutant genes containing mutations for D330A or Y414A were generated and inserted into pMD18-T to produce recombinant plasmids. Next, we used a reverse genetic system to generate MHV-A59 N7-MTase mutant viruses as described previously [26]. Briefly, monolayer CV-1 cells were infected with vMHV-nsp14-gtp-in, followed by transfection of the recombinant plasmids using Lipofectamine 2000 transfection reagent (Invitrogen). Two or three days later, the cells were harvested and recombinant vaccinia viruses were isolated by three rounds of plaque purification with GPT-positive or GPT-negative selection as described previously [27]. GPT-positive clones were selected by three rounds of plaque purification on CV-1 cells in the presence of xanthine, hypoxanthine and mycophenolic acid, while GPT-negative clones, in which the GPT gene was replaced by the nsp14 mutant gene, were selected by three rounds of plaque purification on D980R cells in the presence of 6-thioguanine. Recombinant MHV-A59 nsp14 mutants were rescued on a modified BHK-21 cell line expressing the MHV-A59 N protein. The cDNA of recombinant vaccinia viruses vMHV-inf-1 or vMHV-nsp14-mutant were extracted, purified and cleaved, and the resulting DNA products were used as templates for the transcription of MHV-A59 RNA, which was transfected into BHK-21-MHV-N cells. Following transfection, the transfected BHK-MHV-N cells were mixed with 17Cl-1 cells at a ratio of 1/4. At one to two days post transfection, cell culture supernatants were harvested, and recombinant MHV-A59 N7-MTase mutants D330A and Y414A, as well as recombinant MHV-A59 wild type (WT), were plaque purified three times, and a single plaque was used to produce a virus stock. Viruses were aliquoted and stored at –80°C.

### Viral plaque assay

MHV viruses were amplified on Neuro 2a cells and titrated on L2 cells by plaque assay. Briefly, serial 10-fold dilutions of viruses were added into monolayer L2 cells. After 1 h of adsorption at 37°C, the inoculum was removed and cells were supplemented with DMEM containing 1.0% methylcellulose and 5% FBS. Plates were incubated for 2 days until obvious plaques can be observed. Cells were stained with 1% crystal violet for 12 h. Plaques were counted and viral titres were expressed as PFU/mL.

### Genetic stability of mutants

To examine the genetic stability of mutants, viruses were separately passaged on Neuro 2a cells for 10 rounds. Six-well plates were seeded with Neuro 2a cells and viruses P0 were added to infect Neuro 2a cells. At 16 h post infection (hpi), culture fluids were harvested and transferred to a new well containing fresh Neuro 2a cells. After 10 rounds of such passaging, cell total RNA was extracted and cDNA was obtained using reverse transcription. The DNA segment encoding nsp14 was amplified and the products were subjected to DNA sequencing.

### Virulence of mutants in vivo

For the virulence and pathogenesis study, 4-week-old male SPF C57BL/6 mice were anaesthetized and followed by intrahepatic (i.h.) inoculation with 100 μL of DMEM containing 2×10^6^ PFU MHV-A59 WT virus or N7-MTase mutants, while 100 μL of DMEM was i.h. inoculated as a mock control. Infected mice were monitored for body weight loss, clinical signs of disease and mortality daily. At 1, 2, 5 days post infection (dpi), mice were sacrificed and sera were collected for detection of alanine aminotransferase (ALT) levels according to the manufacturer’s recommendations. Liver was also collected for viral load determination and histological analysis.

### Vaccination and challenge of mice

For immunization, 4-week-old female C57BL/6 mice were subcutaneously vaccinated with a 100 μL volume containing either 5×10^5^ PFU MHV-A59 WT, D330A, Y414A or DMEM mock. At 2 weeks post immunization (wpi) and 4 wpi, sera of infected animals were collected for IgG and neutralization antibody measurement. At 4 wpi, mice were sacrificed and spleens were removed to prepare single-cell suspensions for cytokine assay. To test the duration that neutralizing antibodies persist for, mice were kept for 48 weeks and sera were collected every 4 weeks. Mice were challenged with 2×10^7^ PFU MHV-A59 by i.h. at 30 days post infection (dpi), then body weight and survival were monitored. The liver was then removed for viral titre determination and histological analysis.

### IgG titre measurement

An enzyme-linked immunosorbent assay (ELISA) was conducted to measure the IgG antibody titre against MHV in the sera. Briefly, twofold serially diluted sera were added to 96-well microtiter plates precoated with heat-inactivated MHV-A59. The plates were incubated at 37°C for 2 h, followed by five washes with PBST. After that, AP-conjugated goat anti-mouse IgG antibodies (Southern Biotech) were added and incubated at 37°C for 1 h. Finally, the substrate 4-Nitrophenyl phosphate disodium salt hexahydrate (Sigma) was added to the plates, and 45 min later the absorbance at 405 nm was measured by an ELISA plate reader (Tecan, San Jose, CA). Endpoint titres were defined as the lowest dilution of serum in which binding was twofold greater than the mean binding observed in the negative controls.

### Plaque reduction neutralization test

MHV-specific neutralizing antibody titres in immunized mouse sera were measured by a standard 50% plaque reduction neutralization test (PRNT_50_). Briefly, twofold serial dilutions of heat-inactivated sera were mixed with equal volumes of approximately 100 infectious MHV-A59 and incubated at 37°C for 1 h. The resulting virus–serum mixtures were then transferred to confluent L2 cells, followed by 1 h of incubation. L2 cells were grown in 1.0% methylcellulose medium and stained as described above. Plaques were counted and PRNT_50_ values were calculated by the Reed–Muech method.

### Splenocyte stimulation and cytokine assay

Splenocytes of vaccinated mice were prepared and stimulated with WT MHV virus in vitro and the production of cytokines was analysed by FACS and ELISA. Briefly, approximately 5×10^6^ splenocytes were stimulated with 5×10^6^ live WT MHV-A59. For ELISA, cell supernatants were directly collected at 48 hpi to detect the secretion of interferon-γ (IFN-γ), interleukin-2 (IL-2), IL-10, and IL-4. For intracellular cytokine staining, brefeldin A (Sigma) at a final concentration of 5 ng/mL was added into wells to block protein transport during the final 5 h of stimulation. After that, cells were stained with anti-mouse mAbs CD3-PERCP (Biolegand), CD4-FITC (Biolegend), or CD8-APC (4A Biotech) for 30 min at 4°C, which was followed by treatment with fixation buffer and permeabilization buffer in accordance with the manufacturer’s protocol (Biolegend). Then, either PE-conjugated anti-IFN-γ antibody or an isotype control of rat IgG1 (Biolegand) was added to cells. The stained samples were analysed with flow cytometer FACSCalibur (BD Biosciences) and the data were analysed by FlowJo software.

### Statistical analysis

All experiments were performed at least three times and the results were measured as a mean ± SD or mean ± SEM. Student’s *t*-tests were used to measure statistical differences between samples using GraphPad Prism 7 (GraphPad Software Inc, USA), in which *P* < 0.05 was considered a statistically significant difference.

## Results

### Construction and characterization of MHV N7-MTase mutants

**Figure 1.**
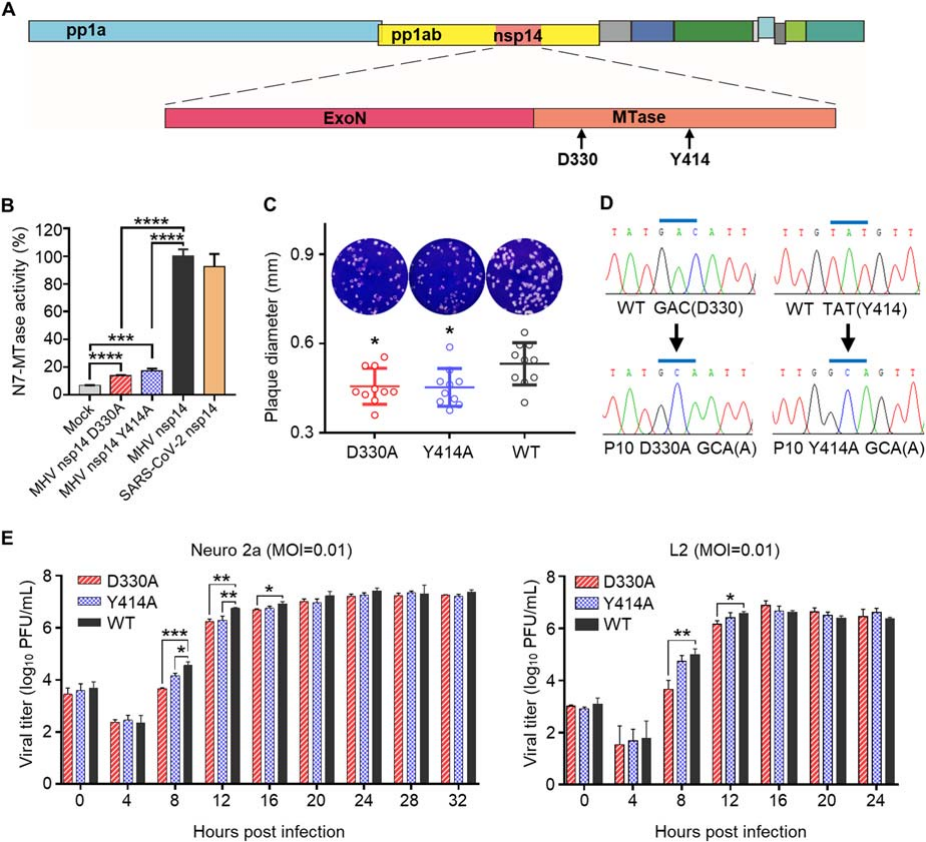
Construction and characterization of MHV nsp14 N7-MTase mutants. (A) Structure of MHV-A59 genome. The mutation sites in nsp14 N7-MTase domain are shown. (B) N7-MTase activity assay for nsp14 and its mutants in vitro. The N7-MTase activity of WT MHV nsp14 was set as 100%. Means with SD are shown (*n* = 3). (C) Plaque morphology of mutant D330A, Y414A and WT virus at passage 10 (P10). Viruses were passaged on Neuro 2a cells for 10 rounds, and the resulting P10 viruses were examined by plaque assay. The diameters of representative plaques were measured and are shown as means with SD (*n* = 10). (D) The D330A and Y414A mutants were maintained after 10 rounds of passage. The cDNA fragments encoding nsp14 of P10 viruses were amplified by RCR and the products were sequenced. (E) One-step replication curves of D330A, Y414A, and WT virus were measured on Neuro 2a cells and L2 cells at an MOI of 0.01. Viral titre in cell supernatant were measured by plaque assay. Means with SD are shown (*n* = 3). ****P < 0.0001; ***P < 0.001; **P < 0.01; *P < 0.05 (unpaired Student’s *t*-test).

In previous studies, we showed that CoV nsp14 possesses guanine N7-MTase activity coupled with known ExoN activity, and the N7-MTase is highly conserved among different CoVs [23,25,28]. Further structure–function analysis suggested that the single amino acid substitution D331A, which is in the DxG S-adenosyl-L-methionine (SAM)-binding motif, and Y420A, which is in the supporting structure of the SAM-binding pocket, can affect the SAM-binding affinity of SARS-CoV N7-MTase and abolish its N7-methylation activity in vitro [24]. Alignment of nsp14 amino acid sequences showed that the two residues are also highly conserved among different CoVs (supplementary Figure S1). To verify the effects of these conserved key residues on MHV N7-MTase enzymatic activity, we introduced the two mutations into MHV-A59 nsp14 and expressed MHV nsp14 mutant protein D330A as well as Y414A in *E. coli* cells (Figure 1(A)), followed by an N7-MTase activity test in biochemical MTase assays as described previously [24]. In this test, ^3^H-labeled methyl donor [^3^H]-SAM was used and the radiolabeled methyl group was transferred to the unlabelled GpppA-RNA by activated MTase. As expected, while the N7-MTase activity of the WT nsp14 was set as 100%, the N7-MTase activity of mutant nsp14 D330A and Y414A was very low, reaching 14% and 17%, respectively, but was significantly higher than the background value (Figure 1(B)), suggesting that the single amino acid substitution D330A and Y414A can partially abolish N7-MTase activity of MHV nsp14. Additionally, we expressed nsp14 of emerging SARS-CoV-2 and determined its N7-MTase activity. The result showed that SARS-CoV-2 nsp14 possesses strong N7-MTase activity (Figure 1(B)), which also indicates that N7-MTase is highly conserved among CoVs.

To further explore the details of these two mutations in terms of coronavirus replication and pathogenicity, we engineered and rescued two MHV N7-MTase mutant viruses, D330A and Y414A, and wild-type (WT) MHV using a vaccinia virus-based reverse genetic system. The rescued mutant viruses P0 were sequenced and it was confirmed that the designed mutation D330A and Y414A were introduced (data not shown). As Figure 1(C) shows, both of the N7-MTase mutant viruses exhibited smaller plaque morphologies compared to those of the WT viruses. Considering the genetic stability of the mutants during viral replication, we continuously passaged the mutant viruses P0 on Neuro 2a cells for 10 rounds and found that D330A and Y414A mutations still remained, while no extra mutation was introduced within the nsp14 gene segment according to the sequencing results of P10 mutants (Figure 1(D)). P10 viruses were therefore used in latter experiments. The one-step growth curves of mutants in mammalian cell line Neuro 2a and L2 showed that the D330A mutant replicated slightly slower than the WT virus during the early stages (4–12 h), but peaked at similar levels to the WT virus. Meanwhile, the Y414A mutant showed a slightly reduced replication capacity in Neuro 2a cells but possessed similar replication kinetics to WT viruses in L2 cells (Figure 1(E)). Taken together, these results suggest that N7-MTase mutant coronaviruses harbouring a single amino acid substitution of D330A or Y414A are stable and slightly attenuated in vitro.

### N7-MTase mutants are highly attenuated and change the delayed IFN-I production in mice

**Figure 2.**
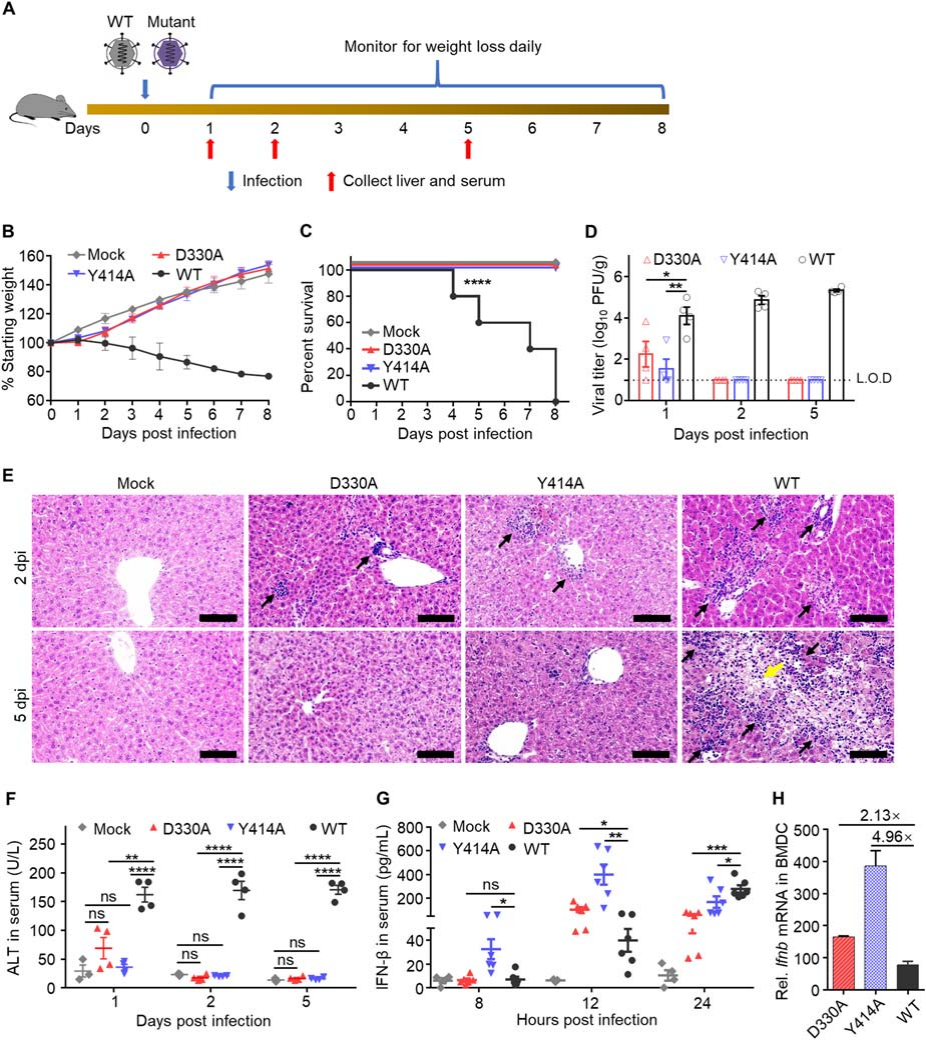
N7-MTase mutants are highly attenuated in vivo. (A) Scheme of infection. Four-week-old C57BL/6 male mice were i.h. infected with 2×10^6^ PFU N7-MTase mutants or WT virus, respectively. An equal volume of DMEM was injected as a mock control. Mice were monitored for 8 days for (B) body weight changes (*n* = 5) and (C) survival (*n* = 5). Means with SD are shown. (D) Viral loads in liver at 1, 2, 5 dpi were measured by plaque assay. Means with SEM are shown (*n* = 4). L.O.D: Limit of detection. (E) Liver histopathological changes of mice. The black arrow indicates inflammatory cell infiltration; the yellow arrow indicates liver fibrosis. Scale bar, 100 μm. (F) ALT levels in mice sera. Liver damage was evaluated by measuring the ALT level in serum. Means with SEM are shown (*n* = 3–4). (G) The IFN-β level in mice sera was detected by ELISA at 8, 12, 24 hpi. Means with SEM are shown (*n* = 3–6). (H) The relative *Ifnb* mRNA level in BMDCs was detected at 8 hpi. Means with SD are shown (*n* = 3). ****P < 0.0001; ***P < 0.001; **P < 0.01; *P < 0.05; ns, not significant, *P* > 0.05 (unpaired Student’s *t*-test).

MHV strain A59 (MHV-A59) can cause acute hepatitis or even death after intraperitoneal inoculation of 4–6-week-old C57BL/6 mice [29,30]. To compare the virulence and pathogenicity of the mutant and WT viruses in vivo, groups of 4-week-old male C57BL/6 mice were intrahepatically inoculated (i.h.) with 2×10^6^ PFU of mutant D330A, Y414A or WT MHV-A59. We then monitored the mice for body weight loss and clinical signs of disease and mortality daily (Figure 2(A)). At the early stage of infection (≤24 h), mice infected with mutant D330A, Y414A or WT suffered a short-term body weight plateau with minor symptoms of illness. However, as the infection progressed, both D330A- and Y414A-infected mice started to gain weight slowly and their survival rate increased compared to WT-infected mice, which lost massive weight until death (Figure 2(B,C)). Furthermore, mice infected with the WT virus presented significantly higher levels of viral titres in the liver at 1, 2, 5 dpi, whereas the viral load of mice infected with D330A and Y414A could only be detected at 1 dpi and manifested as 68-fold and 350-fold decrease compared to WT virus-infected mice, respectively (Figure 2(D)). These results indicate that the mutant D330A and Y414A were sharply replication-limited and were quickly cleared by host animals.

Further examination of liver function and injury also revealed significant attenuation of N7-MTase mutants in mice. Both liver histopathological analysis and alanine aminotransferase (ALT) measurement showed that only minimal signs of disease occurred in mice after infection with the D330A and Y414A mutants at 1 dpi (Figure 2(E,F)). In contrast, mice inoculated with WT viruses maintained high levels of ALT from 1 to 5 dpi, which means that the liver damage was gradually exacerbated. These results were correlated with the greatest loss of body weight (Figure 2(B)) and viral loads (Figure 2(D)). Moreover, as the viral infection persisted, fibrosis and the connective tissues became obvious in the liver of WT groups (Figure 2(F)), while the mock infection group and mutant groups did not show any lesions in the liver at 5 dpi (Figure 2(F)).

Having observed highly attenuated phenotypes of N7-MTase mutants, we next compared the IFN-I production induced by N7-MTase mutants and the WT virus. As Figure 2(G) shows, IFN-β levels in the sera of D330A-infected and Y414A-infected mice accumulated from 8 hpi and peaked at 12 hpi, while the IFN-β levels of WT-infected mice were significantly lower than N7-MTase mutants. At 24 hpi, mutant-infected mice gradually recovered from infection and the amount of IFN-β also declined, whereas WT MHV replicated to higher titres and induced more IFN-β to resist viral infection (Figure 2(D)). Similarly, higher *Ifnb* mRNA levels were induced in mouse bone marrow-derived dendritic cells (BMDCs) at 8 h after infection with mutant D330A (2.13-fold increase) and mutant Y414A (4.96-fold increase) compared to WT virus-infected mice (Figure 2(H)). These results are consistent with the reports that CoVs can delay IFN-I signalling, leading to elevated cytokine/chemokine levels at the late stage of infection after the viral titres peak [31,32]. Interestingly, our results showed that N7-MTase mutants could induce an earlier IFN-I production than the WT virus did in mice, which altered the delayed IFN-I production of CoVs.

### N7-MTase mutants induced both humoral and cellular immune responses in mice

**Figure 3.**
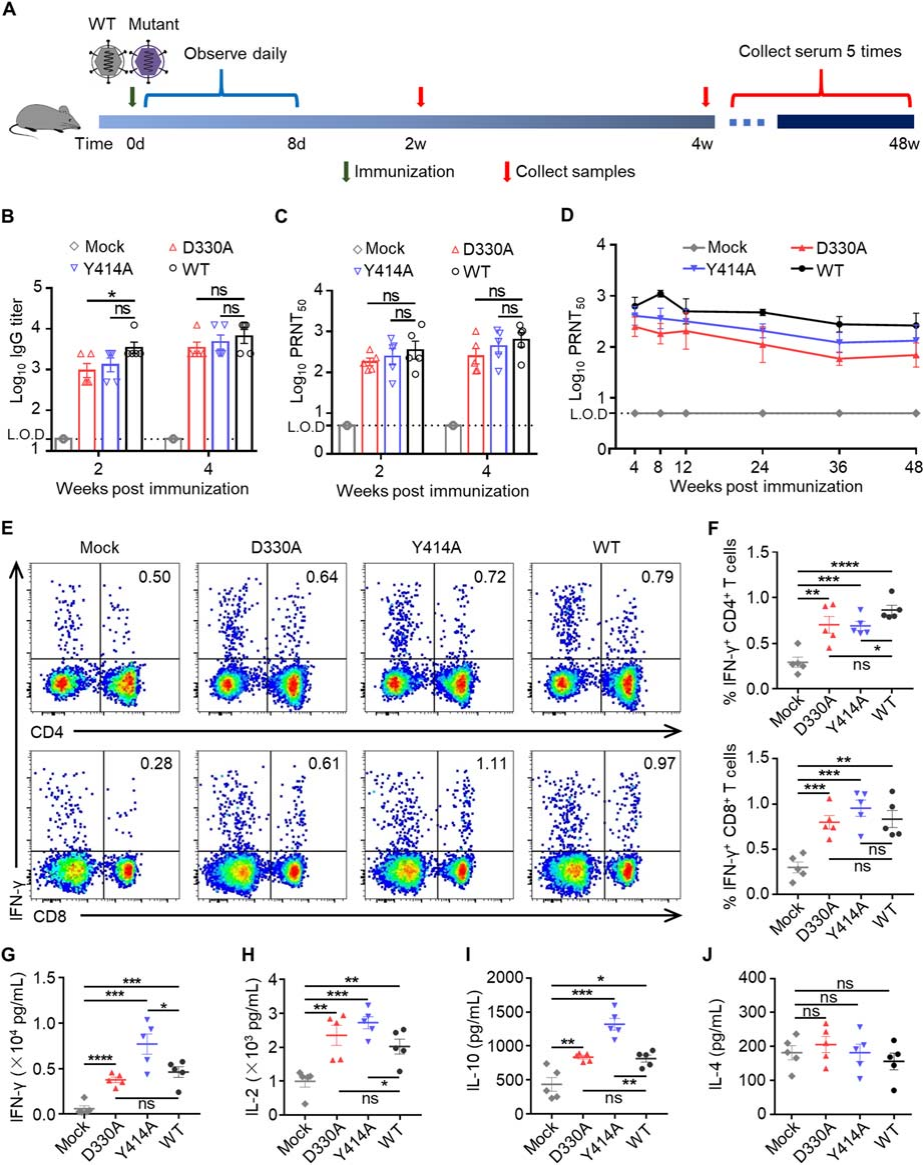
MHV nsp14 N7-MTase mutants induced both humoral and cellular immune responses in mice. (A) Scheme of immunization. Four-week-old C57BL/6 mice were s.c. immunized with 5×10^5^ PFU N7-MTase mutants or WT viruses. Sera were collected at 2 and 4 wpi. At 4 wpi, mouse spleens were harvested. (B) Virus-specific IgG titre in mice serum. Sera were 5-fold serial-diluted and the IgG titre was then measured by ELISA. Means with SEM are shown (*n* = 5). (C) Neutralizing antibody titre in sera of mice. To neutralize the antibody titre, sera were 2-fold serial-diluted to PRNT_50_ test. Means with SEM are shown (*n* = 5). (D) Virus-specific neutralizing antibodies can exist for at least 48 weeks in the sera of immunized animals. Means with SEM are shown (*n* = 3∼5). (E) Spleens of immunized mice were collected at 4 wpi. Single splenocytes were cultured ex vivo and stimulated with WT MHV for 24 h, after which they were stained for markers (IFN-γ, CD3, CD4 or CD8). The T-cells were gated and the percentages of (F) IFN-γ^+^ CD4^+^ cells and (G) IFN-γ^+^ CD8^+^ cells are shown. Means with SEM are shown (n = 5). (G), (H), (I), and (J) Total splenocyte supernatant from the ex vivo culture was collected 48 h after WT MHV stimulation, and IFN-γ, IL-2, IL-10, and IL-4 production were measured by ELISA. Data are presented as means ± S.E.M. Means with SEM are shown (*n* = 5). ****P < 0.0001; ***P < 0.001; **P < 0.01; *P < 0.05; ns, not significant, *P* > 0.05 (unpaired Student’s *t*-test).

Based on the attenuation of N7-MTase mutants in mice, we further evaluated the immunogenicity and protective efficacy of the mutants in mice to investigate the possibility of targeting viral N7-MTase to design live attenuated vaccines. For vaccination, groups of 4-week-old male C57BL/6 mice were subcutaneously (s.c.) inoculated with a dose of 5×10^5^ PFU mutant D330A, Y414A and WT MHV-A59, while DMEM was used as a mock control (Figure 3(A)). Mice were observed for 8 days for symptoms of infection. As expected, this mild inoculation method through the s.c. route did not cause a pathology in mice from both the mutant and WT groups. At 2 and 4 wpi, sera of animals were collected for evaluation of humoral immune responses. The results showed that MHV-specific IgG induced by N7-MTase mutants rapidly reached a comparative level to the WT virus as early as 14 dpi and this could be maintained until 28 dpi (Figure 3(B)). Furthermore, we determined the neutralizing antibody (NA) titres in the serum of immunized animals by PRNT_50_ assay. As expected, the PRNT_50_ values of virus-infected mice rapidly reached a significantly higher level than the mock group at both two and four weeks after immunization (Figure 3(C)). Although strong humoral immune responses were induced, the IgE induced by viruses was as low as the mock group (data not shown), suggesting that N7-MTase mutants did not mediate an anaphylactic reaction. Surprisingly, continuous neutralizing antibody monitoring showed that the NA could last for at least 48 weeks in serum (Figure 3(D)). These results indicated that a robust and long-term humoral immune response was activated after immunization with attenuated N7-MTase mutants in mice.

While antibodies are crucial to reducing the viral load by binding and neutralizing virus particles, T cell responses are also necessary for efficient viral clearance [33-35]. To detect the type and level of T-cell responses, we immunized C57BL/6 mice with both N7-mutants and WT virus, as mentioned above. The spleens of mice were harvested at 4 wpi for restimulation in vitro. We then detected cytokines production through an intracellular cytokine staining assay (ICS) and ELISA. The results showed that CD4^+^ and CD8^+^ T cells of both WT- and mutant-immunized mice had a higher interferon (IFN)-γ response than the mock-immunized group after re-stimulation with WT MHV-A59 (Figure 3(E–G)). Notably, the strength of the T cell response was similar in the D330A, Y414A, and WT groups. Furthermore, total splenocytes induced more Th1 cytokine IFN-γ, interleukin (IL)-2 and Th2 cytokine IL-10 (Figure 3(H,I)) than the mock group. The splenocytes of mice immunized with Y414A produced higher levels of cytokines than cells immunized with the WT virus (IFN-γ, *P* = 0.0399; IL-2, *P* = 0.0388; IL-10, *P* = 0.0010). Taken together, our findings show that coronavirus N7-MTase mutants can induce long-lasting humoral responses, as well as robust CD4^+^ and CD8^+^ T cell responses in mice.

### Immunization with N7-MTase mutants protected C57BL/6 mice from death

**Figure 4.**
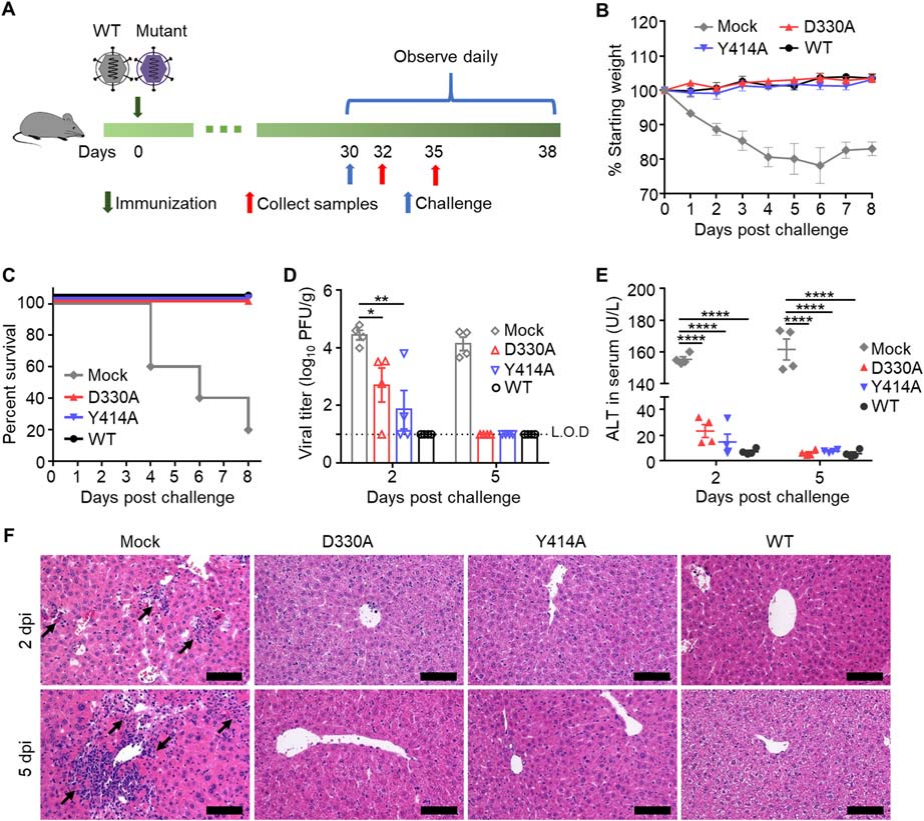
MHV nsp14 N7-MTase mutants immunization protects mice from death after challenge. (A) Scheme of challenge of C57BL/6 mice. Four-week-old C57BL/6 mice were immunized with 5×10^5^ PFU D330A, Y414A, while the WT virus and DMEM were used as positive and mock control, respectively. At 30 dpi, animals were i.h. challenged with a high lethal dose of 2×10^7^ PFU WT MHV. (B) Body weight change. Means with SD are shown (n = 5). (C) Survival 8 days after challenge (*n* = 5). (D) Viral loads in livers were measured by plaque assay at 2- and 5- days post challenge. Means with SEM are shown (*n* = 4). L.O.D: Limit of detection. (E) Liver damage was evaluated by measuring the ALT level in sera. Means with SEM are shown (*n* = 4). (F) Liver histological changes of mice. Black arrows indicate inflammatory cell infiltration. Scale bar, 100 μm. ****P < 0.0001; **P < 0.01; *P < 0.05 (unpaired Student’s *t*-test).

To determine the protective efficacy of N7-MTase mutants, we immunized mice as mentioned above, and then they were challenged with WT MHV-A59 at a lethal dose of 2×10^7^ PFU by the i.h. route (Figure 4(A)). Four of the five mock-immunized mice showed drastic weight loss and died within eight days after the challenge, whereas all the virus-immunized mice survived the lethal challenge (Figure 4(B,C)). Moreover, viral load detection showed that MHV viruses replicated at a minimal level in the liver of all virus-immunized mice at 2 dpi. At 5 dpi, viruses were not detected in the liver of virus-immunized mice, whereas a significantly higher viral titre was maintained in the liver of mock-immunized mice (Figure 4(D)). Similar results were shown in the ALT assay and liver histopathological analysis (Figure 4(E,F)), in which no liver damage was observed after challenge with high pathogenic WT virus. Thus, these results indicated that a single dose of N7-MTase mutant immunization could provide full protection against lethal challenges.

## Discussion

Since the first coronavirus was documented in 1931, an increasing number of CoVs have been discovered from different contagious diseases, which have great impacts on human health and the economy. Although significant progress has been made on the origin of CoV, cross-species transmission, replication, virus–host interactions, and pathogenesis, there is still lack of effective therapeutic options for CoV infection [3]. Thus, vaccination remains the most promising method with which to prevent CoV infection.

Nowadays, available strategies to develop coronavirus vaccines include live attenuated vaccines (LAV), inactivated vaccines, recombinant viral vector vaccines, subunit protein vaccines, DNA vaccines, and RNA vaccines, among others [36]. LAVs generally consist of weakened versions of the pathogens, which can defectively replicate but closely mimic the protective immunity that the live pathogen induces in hosts, hence even a single immunization can elicit strong and long-term adapted immune responses [37]. Successful experience in CoV vaccine development started with livestock CoV prevention in the last century. Live attenuated IBV vaccines and live attenuated TGEV vaccines, which are usually produced by serially passaging pathogenic strains in cells or animals, are most commonly used in all sectors of the poultry and pork industry [38,39]. However, one main concern about LAVs is that there is a risk of reversion to virulence due to their unclear attenuation mechanism and the possibility of their recombination with other circulating CoVs. By contrast, other kinds of vaccines are relatively safer, but have multiple shortcomings such as weak immunogenicity, lack of cytotoxic T lymphocyte (CTL) responses mediated by CD8^+^ T cells, the need for several booster immunization, and higher costs. Considering both the cost and effectiveness, a more safe, affordable, and fully protective LAV would be most desirable.

In this proof-of-concept study, we targeted the core functional domain of CoV N7-MTase and mutated two conserved key activity residues at positions D330 and Y414 of MHV nsp14 to rationally design a live attenuated coronavirus vaccine by the reverse genetic system. In contrast to the traditional method used to develop LAVs by passaging WT viruses on cell lines or animals, this strategy, based on the reverse genetic system, has the advantage of precisely introducing the specific mutation to attenuate the virus [40]. Our results showed that the N7-MTase mutants MHV D330A and Y414A were replicative and stable in the cell culture, which indicates that it would be cheap and easy to prepare these mutants as LAVs in a short time. Moreover, D330A and Y414A can be rapidly cleared within 2 dpi after infecting mice, indicating that these mutants are highly attenuated and safe (Figure 2). More importantly, an N7-MTase live attenuated vaccine can induce long-lasting humoral immune responses mediated by neutralizing antibodies and produce robust CD4^+^ and CD8^+^ T-cell immune responses (Figure 3), which are required for viral clearance from a host. However, considering the susceptibility of CoVs to immunocompromised individuals, further study or optimization of the N7-MTase mutant may be needed.

During viral infection, IFN-I, which can be induced by many kinds of pattern recognition receptors (PRRs), plays an important role in initiating innate immune responses, activating the adaptive immune system and promoting the development of antigen-specific T and B cell responses [41]. Therefore, some ligands of PRRs are used as novel adjuvants to overcome the weak immunogenicity of non-LAVs vaccines and to change the immune response patterns [37,42]. Our results showed that N7-MTase mutants, especially Y414A, can induce higher IFN-I production at the early stage of infection without any adjuvants (Figure 2), and can build strong neutralizing antibody responses and cellular immune responses after immunization (Figure 3). Notably, wild-type or natural CoVs are reported to delay IFN-I signalling [31,43,44], but the mechanism is not yet fully understood. *Case et. al.* reported that N7-MTase deficient MHV (G332A) impairs viral replication, enhances sensitivity to the innate immune response, and reduces viral RNA translation efficiency in cell culture. What’s more, another group showed that N7-MTase deficient PEDV (D350A), which belongs to *Alphacoronavirus*, can induce higher IFN-I and IFN-III responses in cell culture [45,46]. Both these two and our studies indicated that CoV nsp14 N7-MTase contributes to the IFN-I delay during CoV infections. Since N7-MTase is the key enzyme to form the mature 5′-cap structures of the CoV RNAs, we speculate that the earlier IFN-I production induced by N7-MTase deficient CoVs is related to the large amount of unmethylated CoV RNAs, which may be recognized by host’s PRRs like Mda5 or Toll-like receptors [22]. IFN-I is secreted and signals through the type I interferon receptor (IFNAR), followed by the establishment of host cell antiviral state, which might be the reason for the rapid clearance of D330A and Y414A in vivo. However, the detailed mechanism mediating this phenotype warrants further study.

In conclusion, we have demonstrated the feasibility of adopting the highly attenuated recombinant N7-MTase mutant as a potential coronavirus live vaccine approach by in vitro and in vivo characterization of the N7-MTase activity of MHV nsp14. This vaccine approach needs further development, especially considering the great need for a low-cost but high-efficacy vaccine for a variety of infectious diseases caused by CoVs in the livestock industry. Meanwhile, this kind of live attenuated CoVs lacking N7-MTase may have the potential to develop into vaccine strains, which can be used to prepare inactivated vaccines (such as current SARS-CoV-2 inactivated vaccines) on account of their reduced biosafety risks. What’s more, this rational design approach may be applicable to other viruses with defined N7-MTase activity for potential vaccine development.

## Supplementary Material

Supplemental Material

## Data Availability

All data obtained for this study is included in the article.
