# Supplementary figures and images for: Live attenuated coronavirus vaccines deficient in N7-Methyltransferase activity induce both humoral and cellular immune responses in mice
Source: Emerg Microbes Infect. 2021 Aug 18;10(1):1626–37. doi: 10.1080/22221751.2021.1964385 (PMC8381960; doi:10.1080/22221751.2021.1964385)

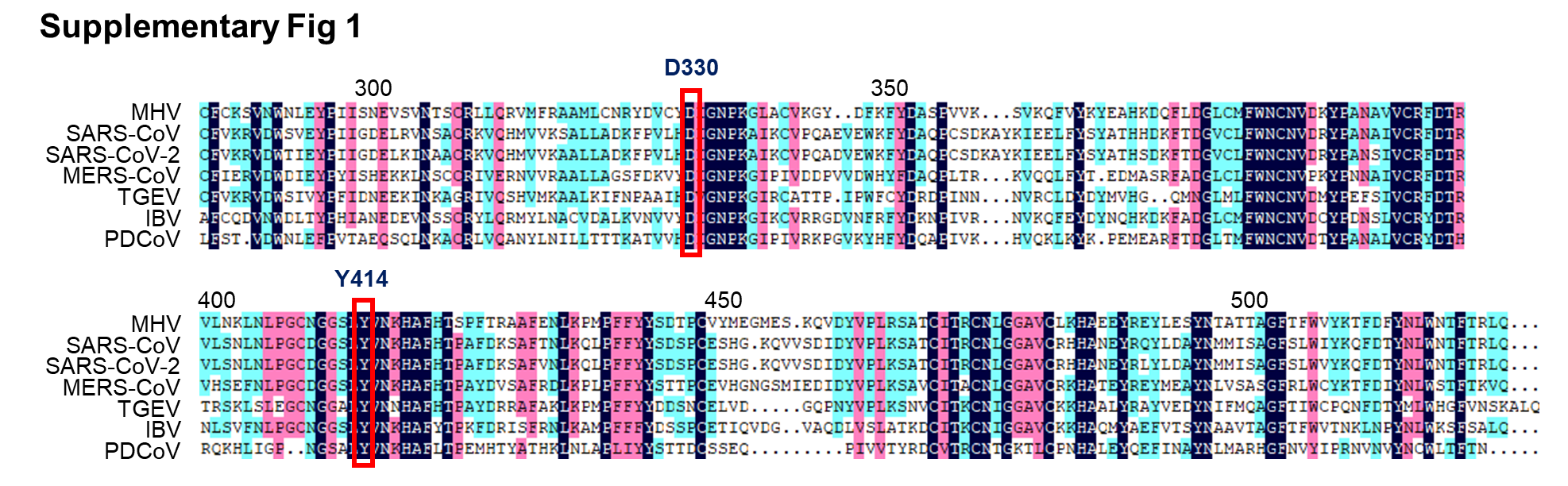

Supplement: Supplemental Material [file TEMI_A_1964385_SM6693.tif]
